# Arterial cardiovascular outcomes and venous thromboembolism in patients with primary Sjögren’s syndrome: a Danish cohort study

**DOI:** 10.1093/rheumatology/keaf210

**Published:** 2025-04-23

**Authors:** Pierre Loiseau, Aurélie Mailhac, Pierre Duhaut, Reimar W Thomsen

**Affiliations:** Department of Clinical Epidemiology, Aarhus University and Aarhus University Hospital, Aarhus, Denmark; Department of Internal Medicine and RECIF, Amiens-Picardie University Hospital, Amiens, France; MP3CV Laboratory, UR UPJV 7517, University of Picardie Jules Verne, Amiens, France; Department of Clinical Epidemiology, Aarhus University and Aarhus University Hospital, Aarhus, Denmark; Department of Internal Medicine and RECIF, Amiens-Picardie University Hospital, Amiens, France; MP3CV Laboratory, UR UPJV 7517, University of Picardie Jules Verne, Amiens, France; Department of Clinical Epidemiology, Aarhus University and Aarhus University Hospital, Aarhus, Denmark

**Keywords:** Primary Sjögren’s syndrome, myocardial infarction, stroke, peripheral artery disease, venous thromboembolism, heart failure, cohort studies

## Abstract

**Objective:**

To evaluate whether atherosclerotic cardiovascular disease outcomes, venous thromboembolism, heart failure, and cardiovascular mortality are elevated in patients with primary Sjögren’s syndrome (pSS) compared with a matched cohort from the general population.

**Methods:**

We conducted a nationwide population-based cohort study including all patients with a pSS diagnosis from 1996 to 2017 in Denmark, matched with a comparison cohort on age, gender, and calendar year. The cumulative incidence of each outcome was computed using hospital contact diagnoses as the end point. We used Cox regression to calculate hazard ratios (HRs) for the endpoints, adjusted for potential confounding factors.

**Results:**

The study included 4697 pSS patients (median age 57 years, 87% women) and 46 970 persons in a comparison cohort followed for a median of 7.6 years (maximum 23 years). After confounder adjustment, pSS was associated with elevated rates of myocardial infarction (adjusted hazard ratio [aHR] 1.23; 95% confidence interval [CI] 1.01–1.50), ischaemic stroke (aHR 1.31; 95% CI 1.14–1.52) and haemorrhagic stroke (aHR 1.51; 95% CI 1.13–2.03), peripheral artery disease (aHR 1.44; 95% CI 1.13–1.83), and venous thromboembolism (aHR 1.57; 95% CI 1.33–1.85). Cardiovascular death (aHR 1.11; 95% CI 0.95–1.30) and heart failure (aHR 1.17; 95% CI 0.99–1.39) were slightly elevated in patients with pSS.

**Conclusion:**

This study provides firm evidence that pSS is associated with increased risk of all atherosclerotic cardiovascular outcomes and venous thromboembolism, and highlights the importance of managing cardiovascular risk factors in these patients.

Rheumatology key messages

- Primary Sjögren's syndrome associates with a wide range of atherosclerotic cardiovascular and venous thromboembolism outcomes.
- We provide first evidence of an elevated peripheral artery disease risk in primary Sjögren’s syndrome.
- An elevated risk of haemorrhagic stroke in primary Sjögren's syndrome is confirmed in this study.

## Introduction

Sjögren’s syndrome (SS) is a systemic autoimmune disease characterized by joint pain, fatigue, and dryness of the mouth and eyes due to local lymphocyte infiltration of the exocrine glands [1]. The syndrome may be a primary disease (primary Sjögren’s syndrome [pSS]) or a secondary disease associated with another autoimmune disease such as rheumatoid arthritis (RA), systemic lupus erythematosus (SLE), inflammatory myopathy, or scleroderma [1, 2].

As for other autoimmune diseases, studies have reported increased arterial stiffness and accelerated atherosclerosis in pSS patients [3]. This may be related to endothelial dysfunction with increased expression of cytokines and pro-inflammatory factors and impaired flow-mediated dilatation, leading to vascular fibrosis and smooth muscle cell proliferation [3–5]. The elevated risk of cardiovascular events documented in large-scale epidemiological studies of RA and SLE suggests that autoimmune diseases should be acknowledged as a clinically important risk factor for cardiovascular disease [6–9].

pSS is a rare condition and evidence of risks of different cardiovascular outcomes in patients with this syndrome is limited and conflicting [10–17]. Most studies have important limitations, including small sample size, short follow-up, absence of differentiation between pSS and secondary SS, and failure to control for confounding factors such as pharmacological treatment. Some cardiovascular diseases, such as peripheral arterial disease, have to our best knowledge never been studied in pSS patients [10–17]. To provide a better understanding of the long-term risk of cardiovascular disease in pSS patients, we investigated the occurrence of myocardial infarction, heart failure, ischaemic and haemorrhagic stroke, peripheral artery disease, venous thromboembolism, and cardiovascular death in a Danish cohort of pSS patients from 1996 to 2018, compared with a matched general population cohort.

## Methods

### Setting and design

The overall setting and design of our cardiovascular outcome analysis has been described previously [18]. In brief, the entire Danish population is covered by a national health care system that provides tax-funded health care. The unique civil registration number (the Central Personal Registry [CPR] number) assigned to all Danish residents at birth or immigration allows linkage of data among national registries [19]. We designed this population-based matched cohort study, using the Danish Civil Registration System, and complete data on hospital-based diagnoses from the Danish National Patient Registry (DNPR). Additionally, we accessed data on all medications prescribed by hospital-based or primary care physicians and dispensed from any community pharmacy in Denmark, as recorded in the Danish National Prescription Registry. The registries do not capture diagnoses from general practices [19]. All codes used in this study are provided in Supplementary Table S1, available at *Rheumatology* online.

### pSS cohort

All patients with a first-time hospital-based discharge diagnosis of pSS recorded in the DNPR from 1 January 1996 to 31 December 2017 were included in the study. They were followed until 31 December 2018, allowing for a minimum of one year of follow-up. The DNPR includes all in-hospital admission records since 1977 and outpatient clinic and emergency department records since 1995. According to the *International Classification of Diseases, Eighth Revision* (ICD-8) (used through 1993) and *Tenth Revision* (ICD-10) (used thereafter), discharge diagnoses are coded with a primary diagnosis (i.e. the main reason for admission) and one or more secondary diagnoses [20]. All recorded inpatient and outpatient diagnoses were used to identify patients with pSS. The index date was defined as the date of the first hospital contact associated with a pSS discharge diagnosis. To avoid inclusion of secondary SS, we excluded all patients with a diagnosis of systemic lupus erythematosus, lupus erythematosus, rheumatoid arthritis, psoriasis, ankylosing spondylitis, systemic scleroderma, dermatomyositis, and polymyositis before the index date. All patients with a history of previous hospital diagnosis of any cardiovascular disease (i.e. myocardial infarction, ischaemic heart disease, stroke [ischaemic or haemorrhagic], cerebrovascular disease, venous thromboembolism, peripheral artery disease, heart failure, and atrial fibrillation or flutter) before the index date were also excluded. This allowed us to focus on first-time incident outcome events.

### General population comparison cohort

We used the Danish Civil Registration System to create a general population comparison cohort [21]. For each patient with pSS, we randomly selected 10 persons without a pSS diagnosis as of the index date and matched them with replacement (i.e. persons in the general population comparison cohort could be matched with more than one pSS patient) on sex, exact age in years, and calendar year of diagnosis. All members of the comparison cohort had to be alive on the index date of their corresponding patient with an incident pSS diagnosis. The general population comparison cohort was subject to the same exclusion criteria as the pSS cohort (i.e. no previous systemic or cardiovascular disease on the index date, as described above).

### Cardiovascular outcomes

Cardiovascular outcomes included first-time myocardial infarction, stroke (ischaemic and haemorrhagic), peripheral artery disease, venous thromboembolism, heart failure, and cardiovascular death (defined as death within 30 days following diagnosis of one of the cardiovascular outcome events). These conditions were identified using all available primary and secondary discharge diagnoses for inpatient and outpatient clinic contacts registered in the DNPR. The accuracy of coding of cardiovascular diagnoses in the DNPR is high, with validation studies consistently reporting positive predictive values >80% for most conditions [20–23]. Unspecified stroke was classified as ischaemic stroke because approximately two-thirds of all unspecified strokes are known to be ischaemic strokes [24]. Among venous thromboembolism events, deep vein thrombosis and pulmonary embolism were analysed as separate outcomes. Moreover, to further investigate the mechanisms underlying venous thromboembolism, unprovoked and provoked events were evaluated separately. Provoked venous thromboembolism was defined as follows: a diagnosis of malignancy at any time prior to the diagnosis of venous thromboembolism; pregnancy; trauma/fracture; or surgery within 90 days before venous thromboembolism (Supplementary Table S2, available at *Rheumatology* online) [22].

### Covariables

The complete hospital history of inpatient and outpatient diagnoses registered in the DNPR was used to obtain information on the following comorbidities recorded before the index date: diabetes, obesity, hyperlipidaemia, hypertension, chronic pulmonary disease, chronic kidney disease, chronic liver disease, alcoholism-related disease, and cancer. We also used the Danish National Prescription Registry to obtain information on medications dispensed in the 180 days prior to the index date. These included medications that allowed us to more completely assess selected comorbidities that may be mainly treated in primary care (diabetes, obesity, hyperlipidaemia, hypertension, chronic pulmonary disease/smoking, and alcoholism-related disease), platelet aggregation inhibitors and anticoagulant therapy, as well as drugs used to treat pSS (corticosteroids, nonsteroidal anti-inflammatory drugs [NSAIDs], and immunosuppressive agents) [23].

### Statistical analyses

The distributions of the key variables and covariables for patients with pSS and the comparison cohort were tabulated. Both cohorts were followed from the index date until the date of myocardial infarction, stroke (ischaemic or haemorrhagic), venous thromboembolism (deep vein thrombosis and pulmonary embolism separately), peripheral artery disease, heart failure, emigration, death, diagnosis of autoimmune disease, or end of follow-up (31 December 2018), whichever occurred first. After an initial event, patients continued to be followed for other subsequent cardiovascular events to avoid informative censoring and to clarify the full spectrum and extent of cardiovascular morbidity associated with pSS. In both cohorts the cumulative incidence per 1000 persons over the entire follow-up period was calculated for each outcome and for the following time periods: 0–1 year, >1–5 years, >5–10 years, >10 years, and 0–23 years, accounting for the competing risk of death using the Aalen-Johansen method [25]. For the same time periods, hazard ratios were computed using stratified (conditional) Cox proportional hazards regression analysis with adjustment for the covariables listed above, except for drugs used for pSS treatment that could be potential mediators or effect measure modifiers.

To provide a better understanding of the cardiovascular risks of pSS, we stratified results for pSS patients *vs* members of the comparison cohort by age, sex, use of NSAIDs, corticosteroids, or immunosuppressive agents, number of cardiovascular risk factors (0, 1, ≥2), type of diagnosis (primary/secondary), and type of hospital contact (inpatient/outpatient). Matching on sex and age was broken when stratifying by drugs used to treat pSS and by number of cardiovascular risk factors (0, 1, ≥2). In these analyses, we adjusted for age and sex instead. Using log-log plots, no deviation was found from the proportionality of hazard assumption in the follow-up periods. All estimated associations are presented with 95% confidence intervals.

### Sensitivity analyses

Several sensitivity analyses were performed. First, to assess if any effect of pSS was dependent on potential pSS therapies, the main analyses were repeated with the addition of the following therapies included in the Cox regression model: NSAIDs, corticosteroids, and immunosuppressive agents. Second, to diminish the risk of selectively more complete confounder adjustment in pSS patients than in comparisons, we performed an analysis excluding any covariates from adjustment that were recorded only within 6 months before the index date, because pre-clinical symptoms of yet undiagnosed pSS may have led to increased recording of cardiovascular disease risk factors in this time window. Third, a two-period analysis (1996–2002 and >2002–2016) was performed, corresponding to the change in diagnostic criteria for pSS in 2002 [26]. This helped us to assess whether the change in diagnostic criteria for pSS may have had an impact on cardiovascular risk outcomes through a change in clinical profile. Fourth, because smoking data were not available in the registries, we assessed the risk of conditions associated with smoking (chronic obstructive pulmonary disease [COPD] and lung cancer) in pSS patients and members of the comparison cohort to evaluate potential differences in underlying smoking prevalence, with the knowledge that pSS may cause chronic bronchitis, but is unlikely to cause lung cancer [1]. Finally, in an additional analysis we assessed 30-day mortality in patients with pSS and comparisons cohort with myocardial infarction, as myocardial infarction prognosis has previously been associated with presence of rheumatological diseases [27, 28]. SAS version 9.4 was used for all statistical analyses. The study was registered at Aarhus University (record number 1880). Under Danish law, patient consents and approvals from ethics committees are not required for noninterventional studies.

### Patient and public involvement

Patients and the public were not involved in the design, conduct, reporting, or plans for disseminating our research.

## Results

**Figure 1.**
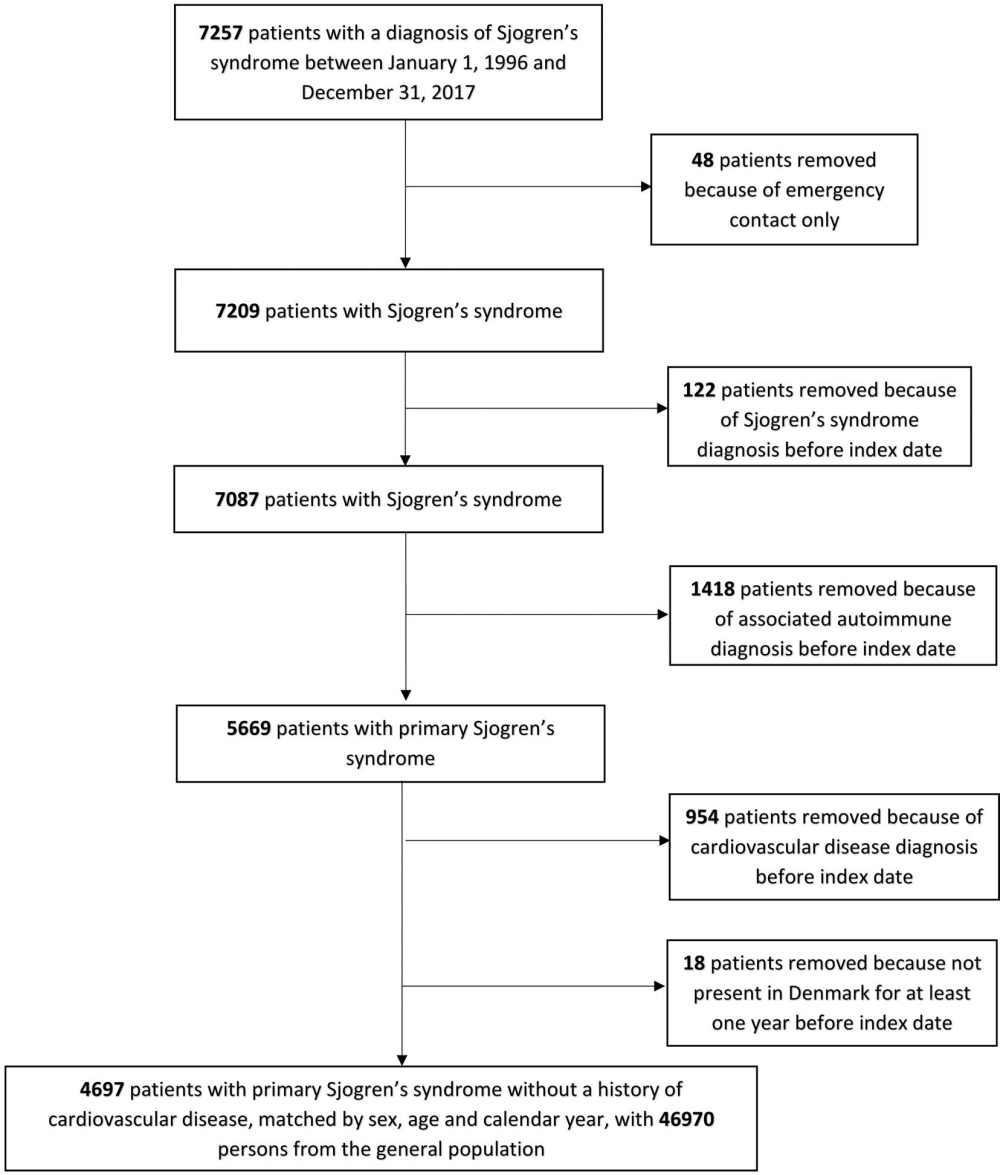
Study flow chart

Fig. 1 shows a flow chart of the study population. After exclusion of patients with emergency contacts only or with an SS diagnosis prior the index date, we identified 7087 patients with an incident diagnosis of SS between January 1, 1996 and December 31, 2017. A total of 1418 (20.0%) of these were excluded because of an associated autoimmune disease. We thus identified 5669 patients with pSS, of whom 954 (16.8%) had a previous history of cardiovascular disease events prior to the index date. An additional post-hoc analysis showed that more than half of these cardiovascular events had occurred within five years of the diagnosis of pSS (Supplementary Fig. S1, available at *Rheumatology* online).

The final study population included 4697 patients with pSS and 46 970 persons in the matched general population comparison cohort (Fig. 1). Median age at pSS diagnosis was 57 years (interquartile range 47–68 years), and 87.2% of the cohort was female. At baseline, patients with pSS had a slightly higher prevalence of cardiovascular risk factors, including diabetes (4.5% in pSS *vs* 4.0% in comparison cohort), hospital-diagnosed obesity (6.3% *vs* 4.4%), or hypertension (18.0% *vs* 15.0%). pSS was also associated with more chronic pulmonary (15.1% *vs* 9.0%), kidney (1.8% *vs* 0.7%), and liver disease (2.8% *vs* 0.7%) and with more previous cancers (9.5% *vs* 6.6%), including known pSS-related cancers such as e.g. lymphoma (0.8% *vs* 0.2% in comparisons). As expected, there was a clearly higher use of medications often used to treat pSS, such as NSAIDs, corticosteroids, and immunosuppressive therapies (Table 1).

**Table 1.** Characteristics of the pSS cohort and the matched general population comparison cohort^a^

Characteristics	Sjogren cohort (*n* = 4697)	General population comparison cohort (*n* = 46970)
Median (interquartile range) age, years	57 (47-68)	57 (47-68)
Women (%)	4097 (87.2)	40 970 (87.2)
Diabetes^b^ (%)	209 (4.5)	1898 (4.0)
Obesity^c^ (%)	294 (6.3)	2083 (4.4)
Hyperlipidaemia^d^ (%)	396 (8.4)	3910 (8.3)
Hypertension^e^ (%)	845 (18.0)	7020 (15.0)
Chronic pulmonary disease^f^ (%)	711 (15.1)	4210 (9.0)
Chronic kidney disease (%)	86 (1.8)	327 (0.7)
Chronic liver disease (%)	129 (2.8)	335 (0.7)
Alcoholism-related disease^g^ (%)	97 (2.1)	767 (1.6)
Cancer (%)	446 (9.5)	3100 (6.6)
Aspirin+ other platelet aggregation inhibitors+ anticoagulant therapy (%)	313 (6.7)	2484 (5.3)
Corticosteroids (%)	438 (9.3)	1217 (2.6)
NSAIDs (%)	1342 (28.6)	6640 (14.1)
Immunosuppressive agents (%)	69 (1.5)	173 (0.4)

a Abbreviation: NSAIDs: Nonsteroidal anti-inflammatory drugs.

b Diagnosis of diabetes/antidiabetic drugs.

c Diagnosis of obesity/redeemed anti-obesity drug prescriptions.

d Diagnosis of hyperlipidaemia or hypercholesterolaemia/redeemed antilipidemic drug prescriptions.

e Diagnosis of hypertension/redeemed prescriptions for at least two antihypertensive drugs.

f Diagnosis of chronic pulmonary disease/redeemed prescriptions for chronic obstructive pulmonary disease.

g Diagnosis of alcohol-related disease/redeemed prescriptions for drugs treating alcohol dependence.

### pSS and cardiovascular disease risk

**Figure 2.**
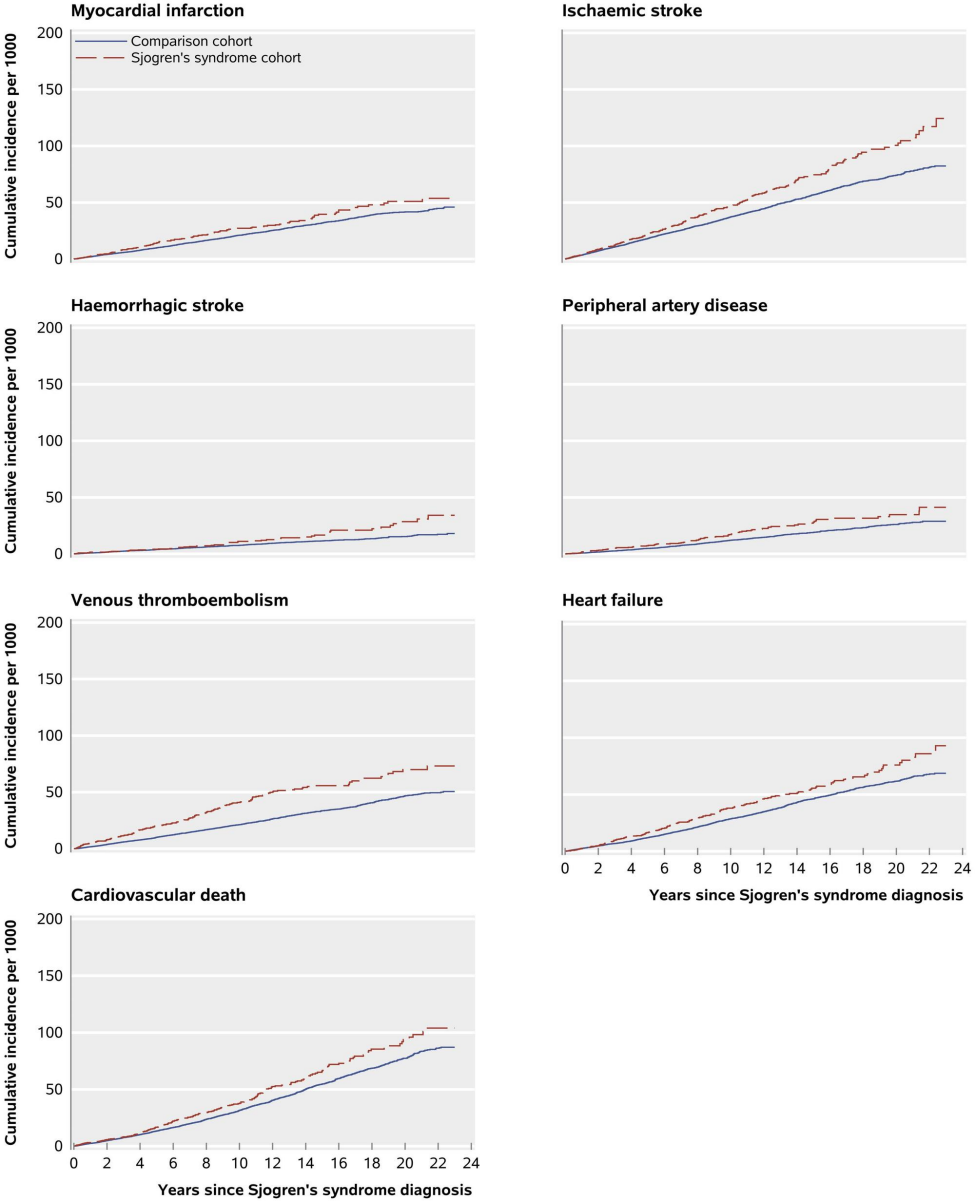
Cumulative incidence of myocardial infarction, ischaemic stroke, haemorrhagic stroke, peripheral artery disease, venous thromboembolism, heart failure, and cardiovascular death among patients with primary Sjögren’s syndrome and members of the comparison cohort

**Figure 3.**
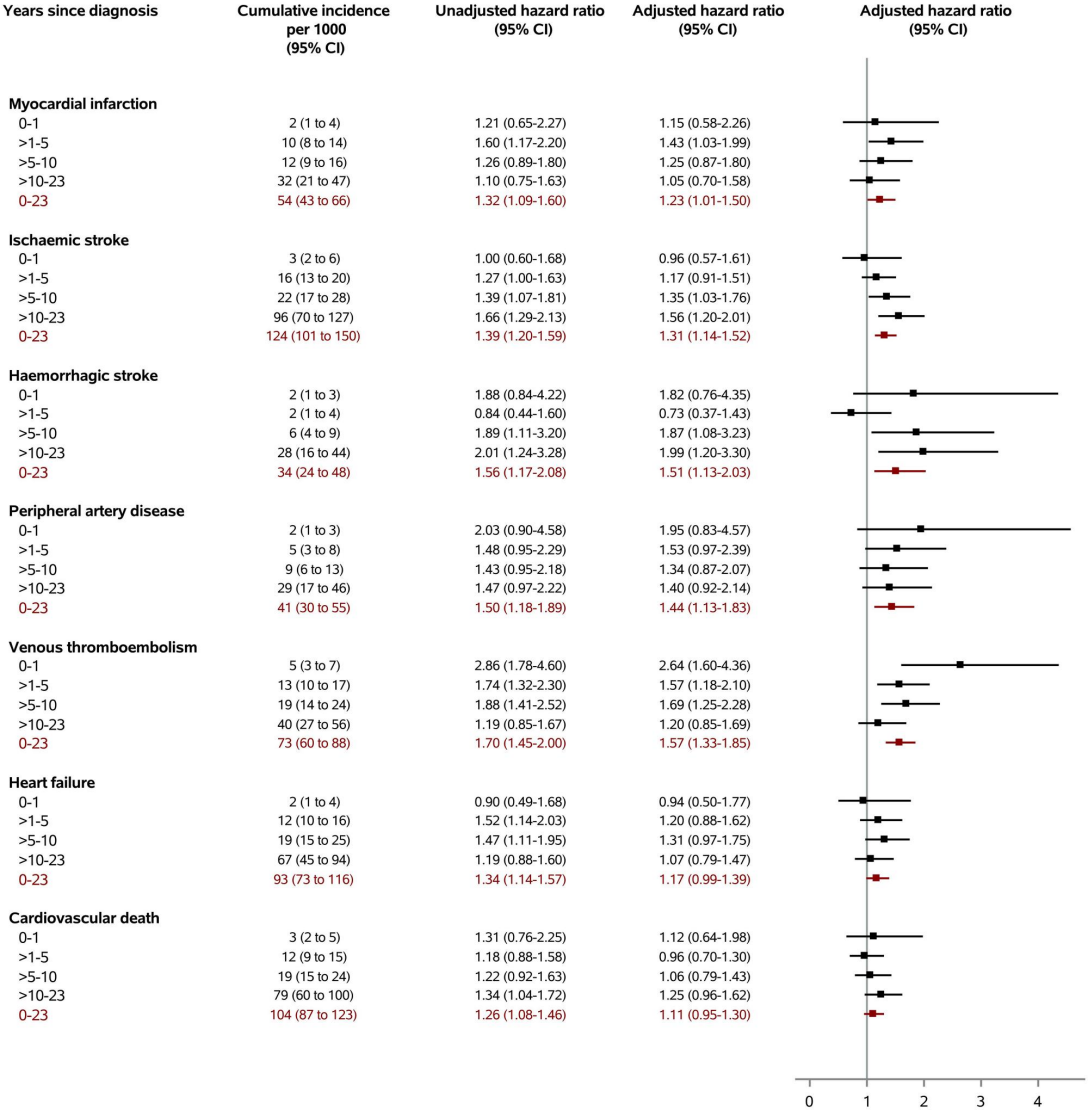
Risk of cardiovascular events among patients with primary Sjögren’s syndrome relative to members of the comparison cohort. Unadjusted hazard ratio: controlled for matching factors (age, sex, and calendar year) by study design. Adjusted hazard ratio: controlled for matching factors (age, sex and calendar year) by study design and adjusted for the covariables in Table 1, except for corticosteroids, NSAIDs, and immunosuppressive agents

The cumulative incidences per 1000 persons in the pSS cohort compared with the general population cohort, over 23 years and with a median follow-up of 7.6 years, were 54 *vs* 46 for myocardial infarction, 124 *vs* 82 for ischaemic stroke, 34 *vs* 18 for haemorrhagic stroke, 41 *vs* 29 for peripheral artery disease, 73 *vs* 51 for venous thromboembolism, and 93 *vs* 69 for heart failure (Fig. 2). After adjustment, pSS was associated with myocardial infarction (adjusted hazard ratio [aHR] 1.23; 95% confidence interval [CI] 1.01–1.50), ischaemic stroke (aHR 1.31; 95% CI 1.14–1.52), haemorrhagic stroke (aHR 1.51; 95% CI 1.13–2.03), peripheral artery disease (aHR 1.44; 95% CI 1.13–1.83), and venous thromboembolism (aHR 1.57; 95% CI 1.33–1.85) (Fig. 3). The relative rates of heart failure (aHR 1.17; 95% CI 0.99–1.39) and cardiovascular death (aHR 1.11; 95% CI 0.95–1.30) also were elevated in pSS patients, although the statistical precision of the estimate was limited. The relative rate of venous thromboembolism appeared to be highest during the first year of follow-up, with a 2.6-fold (95% CI 1.60–4.36) increase, after which aHRs decreased to 1.20 (95% CI 0.85–1.69) after >10 years (Fig. 3). For ischaemic stroke, in contrast, the association with pSS seemed to become higher with follow-up time, with a neutral aHR of 0.96 (95% CI 0.57–1.61) in the first year and an aHR of 1.56 (95% CI 1.20–2.01) after >10 years of follow-up. The aHR increase appeared to be more stable during follow-up for myocardial infarction and peripheral arterial disease (Fig. 3). The relative rate of deep vein thrombosis was higher than for pulmonary embolism, and was similar for provoked and unprovoked venous thromboembolism (Table 2).

**Table 2.** Cumulative incidence of venous thromboembolism in pSS patients, and hazard ratios based on comparisons with the general population cohort^a^

Years since diagnosis	Cumulative incidence per 1000 in the pSS cohort (95% CI)	Adjusted hazard ratio (95% CI)^b^
Deep vein thrombosis		
0-1	2.81 (1.59–4.71)	2.86 (1.45–5.65)
>1-5	9.30 (6.79–12.49)	2.01 (1.40–2.89)
>5-10	9.07 (6.14–13.02)	1.70 (1.11–2.58)
>10-23	23.28 (13.75–36.95)	1.45 (0.92–2.26)
0-23	42.68 (32.85–54.36)	1.80 (1.45–2.23)
Pulmonary embolism		
0-1	1.94 (0.97–3.60)	1.94 (0.86–4.34)
>1-5	4.53 (2.87–6.90)	1.07 (0.66–1.74)
>5-10	10.63 (7.43–14.81)	1.68 (1.10–2.55)
>10-23	19.05 (11.35–30.12)	1.07 (0.66–1.72)
0-23	34.66 (26.42–44.56)	1.35 (1.06–1.72)
Provoked venous thromboembolism		
0-1	2.16 (1.12–3.89)	2.98 (1.39–6.40)
>1-5	5.67 (3.78–8.26)	1.37 (0.86–2.19)
>5-10	8.65 (5.81–12.50)	1.53 (0.99–2.37)
>10-23	21.20 (13.48–31.79)	1.63 (1.05–2.54)
0-23	35.65 (27.59–45.21)	1.60 (1.26–2.03)
Unprovoked venous thromboembolism		
0-1	3.02 (1.75–4.98)	2.96 (1.53–5.74)
>1-5	8.85 (6.41–11.98)	1.93 (1.34–2.78)
>5-10	11.07 (7.78–15.35)	1.82 (1.22–2.71)
>10-23	24.35 (14.15–39.08)	1.26 (0.79–2.01)
0-23	45.66 (35.13–58.14)	1.74 (1.40–2.17)

a Abbreviation: CI, confidence interval.

b Controlled for the matching factors (age, sex, calendar year) by study design and adjusted for the covariables in Table 1, except for corticosteroids, NSAIDs, and immunosuppressive agents.

### Stratified analyses

Stratified by age, the associations with cardiovascular diseases appeared stronger on a relative scale for middle-aged *vs* elderly patients with pSS, except for myocardial infarction (Supplementary Table S3, available at *Rheumatology* online). Specifically for haemorrhagic stroke and venous thromboembolism, aHRs were higher in the youngest individuals (<45 years) with pSS, although with wide confidence intervals (Supplementary Table S3, available at *Rheumatology* online). In general, the relative rates were similar in men and women for the outcome with the highest incidence, but wide confidence intervals were observed for men because 90% of the pSS patients were women (Supplementary Table S4, available at *Rheumatology* online). Among the two-thirds of pSS patients who at baseline were non-users of drugs including corticosteroids, NSAIDs, and immunosuppressive agents, the association of pSS with haemorrhagic stroke and venous thromboembolism was at least as strong as among users of these drugs (Supplementary Table S5, available at *Rheumatology* online). In contrast, aHRs with pSS appeared to be slightly lower for myocardial infarction, ischaemic stroke, and peripheral artery disease among non-users of these drugs (Supplementary Table S5, available at *Rheumatology* online). Cumulative incidences of cardiovascular outcomes increased with the number of cardiovascular risk factors, apart from peripheral artery disease. aHRs were higher in individuals with pSS who had at least two cardiovascular risk factors for ischaemic stroke (aHR 1.55; 95% CI 1.08–2.22), heart failure (aHR 1.60; 95% CI 1.13–2.25), and particularly haemorrhagic stroke (aHR 2.94; 95% CI 1.46–5.93), but not for other cardiovascular events (Supplementary Table S6, available at *Rheumatology* online). Outpatient clinic contacts were identified for 90% of all patients and primary diagnoses of pSS characterized 80% of all patients. The aHRs therefore were close to those in the main analysis. The statistical precision of the aHRs for inpatient admissions and secondary diagnoses was limited, reflecting the small number of individuals affected (Supplementary Tables S7 and S8, available at *Rheumatology* online).

### Sensitivity analyses

The relative rates in individuals in the pSS cohort compared with the general population cohort were lower after adjustment for drugs used to treat pSS. This was observed for all cardiovascular outcomes studied (Supplementary Table S9, available at *Rheumatology* online). Analyses excluding covariates (including drug treatments) that were recorded within 6 months before the index date yielded slightly higher aHRs (with and without adjustment for use of NSAIDs, corticosteroids, and immunosuppressive agents), compared with the main model (Supplementary Table S10, available at *Rheumatology* online). The cumulative incidence of cardiovascular events in the pSS cohort was lower in the 2003–2016 period than in the 1996–2002 period, with the exception of haemorrhagic stroke. The aHRs of ischaemic stroke, venous thromboembolism, and heart failure were approximately the same in the two periods, whereas the aHRs of myocardial infarction (1.10; 95% CI 0.81–1.49) and peripheral artery disease (1.28; 95% CI 0.91–1.81) appeared lower and the aHR of haemorrhagic stroke (1.82; 95% CI 1.21–2.72) appeared higher during the 2003–2016 period (Supplementary Table S11, available at *Rheumatology* online). Finally, the risk of lung cancer was not elevated in the pSS cohort compared with the comparison cohort (aHR 0.82; 95% CI 0.63–1.07), in contrast to the risk of COPD, which was elevated in the pSS cohort (aHR 1.45; 95% CI 1.33–1.59).

Among patients with myocardial infarction during follow-up, death within 30 days post-myocardial infarction occurred in 12 (10%) of 122 pSS patients with myocardial infarction and 139 (13%) of 1054 patients in comparisons cohort with myocardial infarction. This corresponded to a crude mortality RR of 0.75 (95% CI 0.41–1.65), and an age, sex, and Charlson Comorbidity Index adjusted HR of 0.53 (95% CI 0.29–0.96).

## Discussion

In this nationwide matched population-based cohort study, pSS was associated with an elevated risk of most cardiovascular outcomes. In the short term, this elevation was highest for venous thromboembolism, and persisted over the longer term. The elevated rate of ischaemic stroke increased over follow-up time, while the elevated rates of cardiac events and peripheral artery disease remained stable.

### Study strengths and limitations

Our study has several strengths and limitations. Its population-based design with almost complete long-term follow-up substantially reduced the risk of selection and referral bias from place of residence, insurance, income, age-dependent inclusion, or information loss during follow-up. In the DNPR, the positive predictive values of coding are high for myocardial infarction (97%), ischaemic stroke (97%), peripheral artery disease (91%), and venous thromboembolism (88%), but lower for heart failure (80%) [20, 29]. The validity of the pSS discharge diagnosis in the DNPR remains unknown.

Most outcomes considered in our study have severe acute clinical presentations, reducing the likelihood of surveillance bias. The analysis with further adjustment for drug therapies yielded slightly lower HRs related to pSS, suggesting that some of the association between pSS and vascular outcomes could have been driven by adverse effects of these therapies, or, alternatively, by more severe underlying pSS in patients receiving specific treatment of the disease. Our registry data lacked the clinical detail needed to clearly distinguish these possibilities.

Although our analyses were adjusted for a wide range of potential confounders identified from the existing literature, residual confounding from factors such as physical activity, dietary habits, and smoking, as well as the influence of unknown confounders, cannot be excluded. The results showed no excess risk of lung cancer in pSS patients, which could indicate that there was not an excess of smokers in this group. At the same time, the risk of COPD, which is also smoking-related, was higher for pSS patients. However, it has been estimated that 11% of pSS patients have lung damage, including chronic bronchitis [1], and COPD may thus have been increased for other reasons than smoking in pSS. Other studies also suggest that smoking is similarly or less frequent among pSS patients than in the general population [30].

### Comparison with other studies

Two meta-analyses of studies comparing patients with *vs* without pSS published in 2018 reported an elevated risk of coronary heart disease morbidity, with a pooled relative risk of 1.34 (95% confidence interval 1.06–1.68) and 1.30 (95% CI 1.09–1.55) with 5 and 6 included studies, respectively [31, 32]. With the exception of one study [33], previously estimated vascular outcomes in pSS tended to be higher than ours, possibly because of more complete adjustment for comorbidities in our study, our exclusion of secondary Sjögren’s syndrome, and our longitudinal nature of analysis [16, 34, 35].

Results for the specific risks of ischaemic and haemorrhagic stroke have been inconsistent in the literature and limited to a few studies. The results of our study are nonetheless consistent with those of a nationwide Swedish study for the risk of ischaemic stroke (relative risk 1.31; 95% CI 1.02–1.67), though not for the risk of haemorrhagic stroke (relative risk 0.81; 95% CI 0.26–1.90). However, the Swedish study did not separately analyse cases of primary and secondary Sjögren’s syndrome [14].

A meta-analysis suggested an elevated risk of heart failure, but it included only 3 studies with data on heart failure, with significant biases noted [31]. These results are discordant with those of two other studies showing no elevated risk [34, 36]. Our results showed a modest elevated risk of heart failure.

Concerning the risk of venous thromboembolism, two meta-analyses reported an elevated risk of 1.78 (95% CI 1.41–2.24) based on 2 studies and 2.05 (95% CI 1.86–2.27) based on five studies, which is consistent with our findings [31, 37]. However, no data on the separate risks of provoked and unprovoked venous thromboembolism have been published previously.

A Danish cohort study found an elevated risk of heart failure, ischaemic stroke, myocardial infarction, and venous thromboembolism in pSS patients, corroborating our findings. However, the study did not address the separate risks of provoked and unprovoked venous thromboembolism, pulmonary embolism, and deep vein thrombosis, as well as the risks of haemorrhagic stroke and peripheral artery disease [38].

Previous studies of patients with myocardial infarction have suggested increased length of stay and 30-day readmission rates in those with RA and SLE [27, 28], while little is known for pSS and myocardial infarction prognosis. Our study suggests lower, not higher, 30-day mortality in pSS patients with myocardial infarction compared with other myocardial infarction patients. It is unclear if this could be related to increased surveillance or collider bias in pSS patients, and more research is needed on this topic.

Our study adds to current knowledge by including several previously unstudied outcomes such as peripheral arterial disease and provoked and unprovoked venous thromboembolism.

### Potential biological mechanisms

Although the pathophysiological mechanisms underlying the elevated cardiovascular risk in pSS is not fully understood, several hypotheses have been suggested. Some studies reported a significant increase in intima-media thickness in several locations in pSS patients compared with controls [3, 4]. Moreover, the discordant findings of some studies likely reflect that the development of subclinical atherosclerosis is slow. For example, Zardi *et al.* showed a significant correlation between increased carotid intima media thickness and disease duration in pSS patients [39]. Furthermore, Rachapalli *et al.* showed that the ankle-brachial index was not significantly reduced in pSS patients, except among those whose disease duration was over 10 years [40]. This could explain the increasing excess risk of stroke after 10 years of follow-up in our study, but would not apply to other cardiovascular events such as myocardial infarction and peripheral artery disease. Moreover, chronic inflammation related to autoimmune diseases has been shown to induce a pro-thrombotic state by stimulating the coagulation cascade, inhibiting the anticoagulation pathway, and impairing the fibrinolytic process [41–43]. This may explain the elevated risk of venous thromboembolism.

## Conclusion

In this nationwide cohort study, pSS was associated with elevated of myocardial infarction, ischaemic stroke, haemorrhagic stroke, peripheral artery disease, venous thromboembolism, and with modest elevation of heart failure and cardiovascular death. This highlights the importance of undertaking cardiovascular risk assessments in patients with pSS to identify and manage modifiable risk factors.

## Supplementary Material

keaf210_Supplementary_Data

## Data Availability

Danish data protection legislation does not allow sharing of the individual-level personal data used for this study. Requests to access the Danish health registries used in this study may be sent from researchers at authorized research institutions to Statistics Denmark by e-mail (forskningsservice@dst.dk).

## References

[1] Mariette X , Criswell LA. Primary Sjögren’s Syndrome. N Engl J Med 2018;378:931–9. 29514034 10.1056/NEJMcp1702514

[2] Bowman SJ. Primary Sjögren’s syndrome. Lupus 2018;27:32–5. 10.1177/0961203318801673 30452329

[3] Yong WC , Sanguankeo A, Upala S. Association between primary Sjogren’s syndrome, arterial stiffness, and subclinical atherosclerosis: a systematic review and meta-analysis. Clin Rheumatol 2019;38:447–55. 10.1007/s10067-018-4265-1 30178172

[4] Valim V , Gerdts E, Jonsson R et al Atherosclerosis in Sjögren’s syndrome: evidence, possible mechanisms and knowledge gaps. Clin Exp Rheumatol 2016;34:133–42. 26812164

[5] Shoenfeld Y , Gerli R, Doria A et al Accelerated atherosclerosis in autoimmune rheumatic diseases. Circulation 2005;112:3337–47. 10.1161/CIRCULATIONAHA.104.507996 16301360

[6] O’Sullivan M , Bruce IN, Symmons DPM. Cardiovascular risk and its modification in patients with connective tissue diseases. Best Pract Res Clin Rheumatol 2016;30:81–94. 10.1016/j.berh.2016.03.003 27421218

[7] Aviña-Zubieta JA , Choi HK, Sadatsafavi M et al Risk of cardiovascular mortality in patients with rheumatoid arthritis: a meta-analysis of observational studies. Arthritis Rheum 2008;59:1690–7. 10.1002/art.24092 19035419

[8] Yurkovich M , Vostretsova K, Chen W, Aviña-Zubieta JA. Overall and cause-specific mortality in patients with systemic lupus erythematosus: a meta-analysis of observational studies. Arthritis Care Res (Hoboken) 2014;66:608–16. 10.1002/acr.22173 24106157

[9] Lee JJ , Pope JE. A meta-analysis of the risk of venous thromboembolism in inflammatory rheumatic diseases. Arthritis Res Ther 2014;16:435. 10.1186/s13075-014-0435-y 25253302 PMC4207310

[10] Chung WS , Lin CL, Sung FC et al Increased risks of deep vein thrombosis and pulmonary embolism in Sjögren syndrome: a nationwide cohort study. J Rheumatol 2014;41:909–15. 10.3899/jrheum.131345 24692530

[11] Ramagopalan SV , Pakpoor J, Seminog O et al Risk of subarachnoid haemorrhage in people admitted to hospital with selected immune-mediated diseases: record-linkage studies. BMC Neurol 2013;13:176. 10.1186/1471-2377-13-176 24229049 PMC3833635

[12] Ramagopalan SV , Wotton CJ, Handel AE, Yeates D, Goldacre MJ. Risk of venous thromboembolism in people admitted to hospital with selected immune-mediated diseases: record-linkage study. BMC Med 2011;9:1. 10.1186/1741-7015-9-1 21219637 PMC3025873

[13] Zöller B , Li X, Sundquist J, Sundquist K. Risk of subsequent coronary heart disease in patients hospitalized for immune-mediated diseases: a nationwide follow-up study from Sweden. PLoS ONE 2012;7:e33442. 10.1371/journal.pone.0033442 22438933 PMC3306397

[14] Zöller B , Li X, Sundquist J, Sundquist K. Risk of subsequent ischemic and hemorrhagic stroke in patients hospitalized for immune-mediated diseases: a nationwide follow-up study from Sweden. BMC Neurol 2012;12:41. 10.1186/1471-2377-12-41 22708578 PMC3430565

[15] Zöller B , Li X, Sundquist J, Sundquist K. Risk of pulmonary embolism in patients with autoimmune disorders: a nationwide follow-up study from Sweden. Lancet 2012;379:244–9. 10.1016/S0140-6736(11)61306-8 22119579

[16] Yurkovich M , Sayre EC, Shojania K, Avina-Zubieta A. The risk of myocardial infarction and cerebrovascular accident in patients with SjÖGren’s Syndrome: a general population-based cohort study. Ann Rheumatic Dis 2014;73:142–3.

[17] Luni FK , Malik SA, Khan AR et al Risk of ischemic heart disease in patients With Sjögren’s Syndrome. Am J Med Sci 2017;354:395–8. 10.1016/j.amjms.2017.05.001 29078844

[18] Adelborg K , Szépligeti SK, Holland-Bill L et al Migraine and risk of cardiovascular diseases: Danish population based matched cohort study. BMJ 2018;360:k96. 10.1136/bmj.k96 29386181 PMC5791041

[19] Schmidt M , Schmidt SAJ, Adelborg K et al The Danish health care system and epidemiological research: from health care contacts to database records. Clin Epidemiol 2019;11:563–91. 10.2147/CLEP.S179083 31372058 PMC6634267

[20] Schmidt M , Schmidt SAJ, Sandegaard JL et al The Danish National Patient Registry: a review of content, data quality, and research potential. Clin Epidemiol 2015;7:449–90. 10.2147/CLEP.S91125 26604824 PMC4655913

[21] Pedersen CB , Gøtzsche H, Møller JO, Mortensen PB. The Danish Civil Registration System. A cohort of eight million persons. Dan Med Bull 2006;53:441–9. 17150149

[22] Sørensen HT. Venous thromboembolism and the concepts of the incidence and mortality. J Thromb Haemost 2007;5:690–1. 10.1111/j.1538-7836.2007.02414.x 17263789

[23] Rasmussen L , Valentin J, Gesser KM, Hallas J, Pottegård A. Validity of the prescriber information in the Danish National Prescription Registry. Basic Clin Pharmacol Toxicol 2016;119:376–80. 10.1111/bcpt.12610 27098169

[24] Krarup LH , Boysen G, Janjua H, Prescott E, Truelsen T. Validity of stroke diagnoses in a National Register of Patients. Neuroepidemiology 2007;28:150–4. 10.1159/000102143 17478969

[25] Satagopan JM , Ben-Porat L, Berwick M et al A note on competing risks in survival data analysis. Br J Cancer 2004;91:1229–35. 10.1038/sj.bjc.6602102 15305188 PMC2410013

[26] Vitali C , Bombardieri S, Jonsson R et al Classification criteria for Sjögren’s syndrome: a revised version of the European criteria proposed by the American-European Consensus Group. Ann Rheum Dis 2002;61:554–8. 12006334 10.1136/ard.61.6.554 PMC1754137

[27] Mohamed MO , Roddy E, Ya’qoub L et al Acute myocardial infarction in autoimmune rheumatologic disease: a nationwide analysis of clinical outcomes and predictors of management strategy. Mayo Clin Proc 2021;96:388–99. 10.1016/j.mayocp.2020.04.044 33248709

[28] Sagheer S , Deka P, Pathak D et al Clinical outcomes of acute myocardial infarction hospitalizations with systemic lupus erythematosus: an analysis of nationwide readmissions database. Curr Probl Cardiol 2022;47:101086. 10.1016/j.cpcardiol.2021.101086 34936910

[29] Sundbøll Jens , Adelborg Kasper, Munch Troels, et al Positive predictive value of cardiovascular diagnoses in the Danish National Patient Registry: a validation study. BMJ Open 2016;6:e012832. 10.1136/bmjopen-2016-012832 PMC5129042 27864249

[30] Bartoloni E , Alunno A, Valentini V et al The prevalence and relevance of traditional cardiovascular risk factors in primary Sjögren’s syndrome. Clin Exp Rheumatol 2018;36:113–20. 29998823

[31] Beltai A , Barnetche T, Daien C, Lukas C, Gaujoux-Viala C, Combe B et al Cardiovascular morbidity and mortality in primary Sjögren syndrome: a systematic review and meta-analysis. Arthritis Care Res (Hoboken) 2018;72:131–9. 10.1002/acr.23821 30570824

[32] Yong WC , Sanguankeo A, Upala S. Association between primary Sjögren’s syndrome, cardiovascular and cerebrovascular disease: a systematic review and meta-analysis. Clin Exp Rheumatol 2018;36:190–7. 29600936

[33] Chiang CH , Liu CJ, Chen PJ et al Primary Sjögren’s syndrome and the risk of acute myocardial infarction: a nationwide study. Acta Cardiol Sin 2013;29:124–31. 27122696 PMC4804774

[34] Bartoloni E , Baldini C, Schillaci G et al Cardiovascular disease risk burden in primary Sjögren’s syndrome: results of a population-based multicentre cohort study. J Intern Med 2015;278:185–92. 10.1111/joim.12346 25582881

[35] Mofors J , Holmqvist M, Westermark L et al Concomitant Ro/SSA and La/SSB antibodies are biomarkers for the risk of venous thromboembolism and cerebral infarction in primary Sjögren’s syndrome. J Intern Med 2019;286:458–68. 10.1111/joim.12941 31127862 PMC6851863

[36] Lin CY , Chen HA, Chang TW et al Association of primary Sjögren’s syndrome with incident heart failure: a secondary analysis of health claims data in Taiwan. Ther Adv Chronic Dis 2022;13:20406223221078083. 10.1177/20406223221078083 35222904 PMC8874167

[37] Ungprasert P , Srivali N, Kittanamongkolchai W. Risk of venous thromboembolism in patients with Sjögren’s syndrome: a systematic review and meta-analysis. Clin Exp Rheumatol 2015;33:746–50. 26087815

[38] Sun G , Fosbøl EL, Yafasova A et al Long-term risk of heart failure and other adverse cardiovascular outcomes in primary Sjögren’s syndrome. J Intern Med 2023;293:457–69. 10.1111/joim.13595 36507587

[39] Zardi EM , Sambataro G, Basta F, Margiotta DPE, Afeltra AMV. Subclinical carotid atherosclerosis in elderly patients with primary Sjögren syndrome: a duplex Doppler sonographic study. Int J Immunopathol Pharmacol 2014;27:645–51. 10.1177/039463201402700422 25572746

[40] Rachapalli SM , Kiely PD, Bourke BE. Prevalence of abnormal ankle brachial index in patients with primary Sjogren’s syndrome. Clin. Rheumatol 2009;28:587–90. 10.1007/s10067-009-1099-x 19205787

[41] Esmon CT. The interactions between inflammation and coagulation. Br J Haematol 2005;131:417–30. 10.1111/j.1365-2141.2005.05753.x 16281932

[42] Xu J , Lupu F, Esmon CT. Inflammation, innate immunity and blood coagulation. Hamostaseologie 2010;30:5–6. 20162248

[43] Silvariño R , Danza Á, Mérola V et al Venous thromboembolic disease in systemic autoimmune diseases: an association to keep in mind. Autoimmun Rev 2012;12:289–94. 10.1016/j.autrev.2012.05.002 22575365

