# Supplementary material for: Arterial cardiovascular outcomes and venous thromboembolism in patients with primary Sjögren’s syndrome: a Danish cohort study
Source: Rheumatology (Oxford). 2025 Apr 23;64(8):4678–86. doi: 10.1093/rheumatology/keaf210 (PMC12316372; doi:10.1093/rheumatology/keaf210)
Supplement: keaf210_Supplementary_Data [file keaf210_supplementary_data.zip › rhe-24-3025-File007.docx]

**Supplementary Table S1.** ICD-8, ICD-10, and ATC codes used in the study.

|  | **ICD-8** | **ICD-10** | **ATC codes** |
| --- | --- | --- | --- |
| **Autoimmune diseases** |  |  |  |
| Sjogren | 734.9 | M35.0 | N/A |
| Systemic lupus erythematosus | 734.1 | M32 | N/A |
| Lupus erythematosus | 695.4 | L93 | N/A |
| Rheumatoid arthritis | 712.1, 712.3 | M05, M06 | N/A |
| Psoriasis | 696.0, 696.1 | L40 | N/A |
| Ankylosing spondylitis | 712.4 | M45 | N/A |
| Systemic scleroderma | 734.0 | M34 | N/A |
| Dermatomyositis | 716.0 | M33.0, M33.1, M33.9 | N/A |
| polymyositis | 716.1 | M33.2, M33.9 | N/A |
| **Cardiovascular events** |  |  |  |
| Myocardial infarction | 410 | I21, I22, I23 | N/A |
| Ischaemic stroke | 433, 434 | I63, I64 | N/A |
| Haemorrhagic stroke | 430-432 | I60, I61 | N/A |
| Peripheral arterial disease | 443.8, 443.9 | I73.9 | N/A |
| Atrial fibrillation or atrial flutter | 427.93, 427.94 | I48 | N/A |
| Deep vein thrombosis | 451.00 | I80.1-80.3 | N/A |
| Pulmonary embolism | 450 | I26 | N/A |
| Heart failure | 427.0, 427.1 | I11.0, I13.0, I13.2, I50 | N/A |
| Chronic ischaemic heart disease | 412 | I25 | N/A |
| Cerebrovascular diseases | 430-437 | I60-69, G45, G46 | N/A |
| **Covariates** |  |  |  |
| Hypertension* | 400-404 | I10-I15 | C02, C03A, C03B, C03D, C03EA, C07, C08, C09 |
| Obesity | 277 | E65, E66, E68 | A08 |
| Hyperlipidaemia | 272.01, 272.08, 272.09, 279.01  272.00, 279.00 | E78.1-E78.5  E78.0 | C10AA, C10BA, C10BX |
| Diabetes | 249-250 | E10-E14 | A10A, A10B |
| Cancer | 140-209 | C00-86, C88, C90-96 | N/A |
| Chronic kidney disease | 249.02, 250.02, 581-584, 590.09, 593.20, 753.1 | E10.2, E11.2, E12.2, E13.2, E14.2, I12, I13, N02-07, N11, N14, N18, N19, Q61 | N/A |
| Chronic liver disease | 571 | B18, I85, K70.0-70.4, K70.9, K71, K72.1, K72.9, K73, K74, K76.0, K76.6 | N/A |
| Chronic pulmonary disease | 490-493, 515-518 | J40-47, J60-67, J68.4, J70.1, J70.3, J84.1, J92.0, J96.1, J98.2, J98.3 | R03A, R03B |
| Alcohol-related disease | 291, 303, 571.09, 571.10, 577.10 | E24.4, F10.1-9, G31.2, G62.1, G72.1, I42.6, K29.2, K70, K86.0, L278A, R78.0, Z71.4,  Z72.1 | N07BB |
| **Comedications** |  |  |  |
| Aspirin+ other platelet aggregation inhibitors+ anticoagulant therapy | N/A | N/A | B01AC01, B01AC04, B01AC06, B01AC07, B01AC22, B01AC24, B01AC30, N02BA01, B01AA, B01AB, B01AF, B01AX05 |
| Corticosteroids | N/A | N/A | H02AB |
| Nonsteroidal anti-inflammatory drugs | N/A | N/A | M01A |
| Immunosuppressive agents | N/A | N/A | L01BA01, L04AX03  L04AX01  L04AA13  L01AA01  L04AA06 |

*Definition of hypertension: one ICD-10 code, two or more ATC codes, or a combination of treatment defined by codes C02L, C02N, C03EA, C07B, C07C, C07D, C07E, C07F, C08G, C09B, and C09D.
