# Supplementary material for: Arterial cardiovascular outcomes and venous thromboembolism in patients with primary Sjögren’s syndrome: a Danish cohort study
Source: Rheumatology (Oxford). 2025 Apr 23;64(8):4678–86. doi: 10.1093/rheumatology/keaf210 (PMC12316372; doi:10.1093/rheumatology/keaf210)
Supplement: keaf210_Supplementary_Data [file keaf210_supplementary_data.zip › rhe-24-3025-File008.docx]

**Supplementary Table S2.** Definition of provoked and unprovoked venous thromboembolism. “Provoked” venous thromboembolism is defined as patients with a hospital discharge of malignancy (any time before the venous thromboembolism diagnosis), trauma/fracture, surgery, or pregnancy/delivery within 90 days preceding the date of venous thromboembolism diagnosis.

| **Disease** | **ICD-8** | **ICD-10** | **Other codes** |
| --- | --- | --- | --- |
| **Cancer** (any previous diagnosis; including polycythemia vera and essential thrombocythemia) | 140-209, 287.10 | C00-C99, D45, D47.3 |  |
| **Fracture/Trauma** within previous 90 days | 800.929, 950-959 | S00-T14 |  |
| **Pregnancy** within previous 90 days | 630-680 | O00-O99 |  |
| **Surgery** within previous 90 days |  |  | Previous Danish Classification: 000000-99960; NCSP classification: KA-KQ, KX, KY |
