# Supplementary material for: Arterial cardiovascular outcomes and venous thromboembolism in patients with primary Sjögren’s syndrome: a Danish cohort study
Source: Rheumatology (Oxford). 2025 Apr 23;64(8):4678–86. doi: 10.1093/rheumatology/keaf210 (PMC12316372; doi:10.1093/rheumatology/keaf210)
Supplement: keaf210_Supplementary_Data [file keaf210_supplementary_data.zip › rhe-24-3025-File009.docx]

| **Supplementary Table S3.** Cumulative incidence of cardiovascular events in pSS patients and hazard ratios compared with the general population cohort, by age group. | | | | | | |
| --- | --- | --- | --- | --- | --- | --- |
|  | **Age <45 years** | | **Age 45-65 years** | | **Age >65 years** | |
| **Cardiovascular event** | **Cum. incidence per 1000 in pSS cohort (95% CI)** | **Adjusted hazard ratio (95% CI)*** | **Cum. incidence per 1000 in pSS cohort (95% CI)** | **Adjusted hazard ratio (95% CI)*** | **Cum. incidence per 1000 in pSS cohort (95% CI)** | **Adjusted hazard ratio (95% CI)*** |
| **Myocardial infarction** | 13.73 (4.51 to 33.25) | 0.97 (0.37 to 2.54) | 51.68 (36.09 to 71.20) | 1.17 (0.87 to 1.58) | 85.95 (62.45 to 114.03) | 1.31 (0.99 to 1.72) |
| **Ischaemic stroke** | 23.91 (10.47 to 47.03) | 1.27 (0.62 to 2.57) | 134.00 (98.29 to 175.28) | 1.40 (1.13 to 1.73) | 178.14 (138.19 to 222.29) | 1.23 (1.01 to 1.51) |
| **Haemorrhagic stroke** | 20.29 (4.53 to 59.94) | 2.36 (0.85 to 6.58) | 37.20 (21.81 to 58.91) | 1.56 (1.02 to 2.37) | 36.05 (22.29 to 54.86) | 1.38 (0.88 to 2.17) |
| **Peripheral arterial disease** | 3.93 (0.76 to 14.00) | 1.06 (0.20 to 5.45) | 51.79 (33.79 to 75.22) | 1.52 (1.11 to 2.09) | 47.96 (30.09 to 71.84) | 1.40 (0.96 to 2.03) |
| **Venous thromboembolism** | 42.84 (25.50 to 66.88) | 2.01 (1.20 to 3.36) | 79.13 (59.79 to 101.84) | 1.67 (1.31 to 2.13) | 83.32 (59.77 to 111.69) | 1.35 (1.04 to 1.75) |
| **Heart failure** | 9.46 (2.66 to 25.64) | 1.14 (0.26 to 5.07) | 84.27 (60.73 to 112.54) | 1.24 (0.94 to 1.64) | 198.85 (91.99 to 335.08) | 1.14 (0.92 to 1.41) |
| *Controlled for the matching factors (age, sex, calendar year) by study design and adjusted for the covariables in Table 1, except for corticosteroids, NSAIDs, and immunosuppressive agents.  Abbreviation: CI, confidence interval | | | | | | |
