# Supplementary material for: Arterial cardiovascular outcomes and venous thromboembolism in patients with primary Sjögren’s syndrome: a Danish cohort study
Source: Rheumatology (Oxford). 2025 Apr 23;64(8):4678–86. doi: 10.1093/rheumatology/keaf210 (PMC12316372; doi:10.1093/rheumatology/keaf210)
Supplement: keaf210_Supplementary_Data [file keaf210_supplementary_data.zip › rhe-24-3025-File010.docx]

| **Supplementary Table S4.** Cumulative incidence of cardiovascular events in pSS patients and hazard ratios compared with the general population cohort, by sex. | | | | |
| --- | --- | --- | --- | --- |
|  | **Cumulative Incidence per 1000 in pSS cohort (95% CI)** | | **Adjusted hazard ratio (95% CI)*** | |
| **Cardiovascular event** | **Women** | **Men** | **Women** | **Men** |
| **Myocardial infarction** | 51.18 (39.51 to 64.93) | 69.77 (42.53 to 105.92) | 1.26 (1.01 to 1.56) | 1.21 (0.77 to 1.89) |
| **Ischaemic stroke** | 125.25 (100.90 to 152.33) | 114.44 (62.37 to 183.88) | 1.32 (1.13 to 1.54) | 1.25 (0.84 to 1.86) |
| **Haemorrhagic stroke** | 37.36 (25.44 to 52.71) | 10.47 (3.32 to 26.22) | 1.60 (1.17 to 2.17) | 0.77 (0.26 to 2.31) |
| **Peripheral arterial disease** | 44.04 (31.56 to 59.51) | 20.62 (9.79 to 38.51) | 1.56 (1.21 to 2.02) | 0.82 (0.40 to 1.70) |
| **Venous thromboembolism** | 71.95 (58.06 to 87.73) | 79.65 (52.32 to 114.20) | 1.46 (1.22 to 1.74) | 2.59 (1.68 to 4.00) |
| **Heart failure** | 94.41 (72.85 to 119.25) | 79.97 (44.82 to 128.08) | 1.23 (1.03 to 1.47) | 0.87 (0.54 to 1.40) |
| *Controlled for the matching factors (age, calendar year) by study design and adjusted for the covariables in Table 1, except for corticosteroids, NSAIDs, and immunosuppressive agents.  Abbreviation: CI, confidence interval | | | | |
