# Supplementary material for: Arterial cardiovascular outcomes and venous thromboembolism in patients with primary Sjögren’s syndrome: a Danish cohort study
Source: Rheumatology (Oxford). 2025 Apr 23;64(8):4678–86. doi: 10.1093/rheumatology/keaf210 (PMC12316372; doi:10.1093/rheumatology/keaf210)
Supplement: keaf210_Supplementary_Data [file keaf210_supplementary_data.zip › rhe-24-3025-File011.docx]

| **Supplementary Table S5.** Cumulative incidence of cardiovascular events in pSS patients and hazard ratios compared with the general population cohort, by use of pSS therapies (corticosteroids, NSAIDs and Immunosuppressive agents). | | | | |
| --- | --- | --- | --- | --- |
|  | **Cumulative incidence per 1000 in pSS cohort (95% CI)** | | **Adjusted hazard ratio (95% CI)*** | |
| **Cardiovascular event** | **No pSS therapies** | **pSS therapies** | **No pSS therapies** | **pSS therapies** |
| **Myocardial infarction** | 45.62 (32.66 to 61.67) | 67.30 (49.28 to 89.00) | 1.05 (0.82 to 1.36) | 1.33 (0.99 to 1.78) |
| **Ischaemic stroke** | 123.43 (92.45 to 159.11) | 124.98 (94.80 to 159.51) | 1.16 (0.97 to 1.38) | 1.52 (1.22 to 1.90) |
| **Haemorrhagic stroke** | 30.65 (20.44 to 44.05) | 42.52 (19.92 to 78.36) | 1.61 (1.14 to 2.27) | 1.20 (0.72 to 1.99) |
| **Peripheral arterial disease** | 29.46 (18.42 to 44.59) | 62.89 (39.44 to 93.75) | 1.05 (0.76 to 1.45) | 1.83 (1.29 to 2.61) |
| **Venous thromboembolism** | 75.13 (58.51 to 94.36) | 68.97 (51.08 to 90.35) | 1.68 (1.38 to 2.06) | 1.30 (0.99 to 1.70) |
| **Heart failure** | 87.34 (66.91 to 111.04) | 100.35 (62.71 to 148.25) | 1.08 (0.88 to 1.33) | 1.23 (0.96 to 1.58) |
| *Adjusted for age, sex, and covariables in Table 1, except for corticosteroids, NSAIDs and immunosuppressive agents.  Abbreviation: CI, confidence interval | | | | |
