# Supplementary material for: Arterial cardiovascular outcomes and venous thromboembolism in patients with primary Sjögren’s syndrome: a Danish cohort study
Source: Rheumatology (Oxford). 2025 Apr 23;64(8):4678–86. doi: 10.1093/rheumatology/keaf210 (PMC12316372; doi:10.1093/rheumatology/keaf210)
Supplement: keaf210_Supplementary_Data [file keaf210_supplementary_data.zip › rhe-24-3025-File012.docx]

| **Supplementary Table S6.** Cumulative incidence of cardiovascular events in pSS patients, and hazard ratios compared with the general population cohort, by cardiovascular risk factor status. | | | | | | |
| --- | --- | --- | --- | --- | --- | --- |
|  | **No cardiovascular risk factors**** | | **1 cardiovascular risk factors** | | **≥2 cardiovascular risk factors** | |
| **Cardiovascular event** | **Cum. Incidence per 1000 in pSS cohort (95% CI)** | **Adjusted hazard ratio (95% CI)*** | **Cum. Incidence per 1000 in pSS cohort (95% CI)** | **Adjusted hazard ratio (95% CI)*** | **Cum. Incidence per 1000 in pSS cohort (95% CI)** | **Adjusted hazard ratio (95% CI)*** |
| **Myocardial infarction** | 43.96 (31.55 to 59.33) | 1.17 (0.90 to 1.53) | 67.77 (46.65 to 94.08) | 1.43 (1.03 to 1.99) | 102.28 (41.81 to 194.47) | 1.07 (0.66 to 1.73) |
| **Ischaemic stroke** | 103.75 (81.26 to 129.35) | 1.33 (1.11 to 1.60) | 133.96 (98.00 to 175.58) | 1.19 (0.93 to 1.53) | 448.67 (26.32 to 838.12) | 1.55 (1.08 to 2.22) |
| **Haemorrhagic stroke** | 31.41 (18.48 to 49.76) | 1.37 (0.94 to 2.00) | 34.91 (18.47 to 59.61) | 1.32 (0.76 to 2.30) | 57.15 (15.62 to 139.51) | 2.94 (1.46 to 5.93) |
| **Peripheral arterial disease** | 37.99 (23.70 to 57.36) | 1.48 (1.07 to 2.05) | 49.03 (33.82 to 68.24) | 1.67 (1.14 to 2.42) | 41.48 (14.34 to 92.00) | 0.66 (0.33 to 1.31) |
| **Venous thromboembolism** | 56.62 (42.43 to 73.60) | 1.59 (1.27 to 1.99) | 99.22 (72.76 to 130.42) | 1.70 (1.29 to 2.25) | 136.55 (65.64 to 233.27) | 1.71 (1.15 to 2.56) |
| **Heart failure** | 54.90 (39.70 to 73.48) | 1.08 (0.84 to 1.38) | 144.09 (104.52 to 189.79) | 1.21 (0.93 to 1.57) | 335.28 (56.09 to 660.64) | 1.60 (1.13 to 2.25) |

*Adjusted for age, sex and covariable in table 1, except for corticosteroids, NSAIDs and immunosuppressive agents.

**Cardiovascular risk factors included diabetes, obesity, hyperlipidemia, hypertension, chronic pulmonary disease, chronic kidney disease

Abbreviations: CI, confidence interval
