# Supplementary material for: Arterial cardiovascular outcomes and venous thromboembolism in patients with primary Sjögren’s syndrome: a Danish cohort study
Source: Rheumatology (Oxford). 2025 Apr 23;64(8):4678–86. doi: 10.1093/rheumatology/keaf210 (PMC12316372; doi:10.1093/rheumatology/keaf210)
Supplement: keaf210_Supplementary_Data [file keaf210_supplementary_data.zip › rhe-24-3025-File013.docx]

| **Supplementary Table S7.** Cumulative incidence of cardiovascular events in pSS patients and hazard ratios compared with the general population cohort, by diagnosis type. | | | | |
| --- | --- | --- | --- | --- |
|  | **Cumulative Incidence per 1000 in pSS cohort (95% CI)** | | **Adjusted hazard ratio (95% CI)*** | |
| **Cardiovascular event** | **Primary diagnosis** | **Secondary diagnosis** | **Primary diagnosis** | **Secondary diagnosis** |
| **Myocardial infarction** | 53.82 (40.97 to 69.08) | 54.81 (37.18 to 77.19) | 1.18 (0.94 to 1.48) | 1.44 (0.97 to 2.14) |
| **Ischaemic stroke** | 129.01 (101.39 to 160.04) | 108.29 (74.88 to 148.59) | 1.37 (1.16 to 1.60) | 1.13 (0.82 to 1.56) |
| **Haemorrhagic stroke** | 33.77 (21.83 to 49.71) | 35.14 (16.10 to 66.19) | 1.39 (1.00 to 1.93) | 2.16 (1.12 to 4.17) |
| **Peripheral arterial disease** | 42.73 (29.41 to 59.69) | 35.57 (21.24 to 55.59) | 1.41 (1.07 to 1.84) | 1.60 (0.95 to 2.71) |
| **Venous thromboembolism** | 70.20 (56.03 to 86.41) | 82.84 (56.27 to 115.79) | 1.53 (1.27 to 1.84) | 1.77 (1.23 to 2.55) |
| **Heart failure** | 90.63 (66.99 to 118.54) | 104.18 (77.90 to 134.78) | 1.09 (0.89 to 1.32) | 1.43 (1.04 to 1.96) |
| *Controlled for the matching factors (age, sex, calendar year) by study design and adjusted for the covariables in Table 1, except for corticosteroids, NSAIDs, and immunosuppressive agents.  Abbreviation: CI, confidence interval | | | | |
