# Supplementary material for: Arterial cardiovascular outcomes and venous thromboembolism in patients with primary Sjögren’s syndrome: a Danish cohort study
Source: Rheumatology (Oxford). 2025 Apr 23;64(8):4678–86. doi: 10.1093/rheumatology/keaf210 (PMC12316372; doi:10.1093/rheumatology/keaf210)
Supplement: keaf210_Supplementary_Data [file keaf210_supplementary_data.zip › rhe-24-3025-File014.docx]

| **Supplementary Table S8.** Cumulative incidence of cardiovascular events in pSS patients and hazard ratios compared with the general population cohort, by patient type. | | | | |
| --- | --- | --- | --- | --- |
|  | **Cumulative Incidence per 1000 in pSS cohort (95% CI)** | | **Adjusted hazard ratio (95% CI)*** | |
| **Cardiovascular event** | **Outpatients** | **Inpatients** | **Outpatients** | **Inpatients** |
| **Myocardial infarction** | 53.84 (42.04 to 67.64) | 53.87 (28.63 to 90.58) | 1.23 (1.00 to 1.51) | 1.21 (0.68 to 2.16) |
| **Ischaemic stroke** | 127.66 (102.46 to 155.71) | 95.81 (59.30 to 142.68) | 1.33 (1.15 to 1.55) | 1.18 (0.76 to 1.82) |
| **Haemorrhagic stroke** | 35.94 (24.17 to 51.23) | 20.20 (8.21 to 42.00) | 1.56 (1.14 to 2.12) | 1.03 (0.35 to 3.04) |
| **Peripheral arterial disease** | 41.23 (29.07 to 56.50) | 41.21 (19.45 to 75.66) | 1.39 (1.08 to 1.79) | 2.01 (0.94 to 4.33) |
| **Venous thromboembolism** | 72.78 (58.97 to 88.43) | 78.02 (47.68 to 117.90) | 1.55 (1.30 to 1.85) | 1.71 (1.05 to 2.78) |
| **Heart failure** | 94.07 (72.09 to 119.50) | 88.70 (55.96 to 130.63) | 1.14 (0.95 to 1.37) | 1.40 (0.87 to 2.24) |
| *Controlled for the matching factors (age, sex, calendar year) by study design and adjusted for the covariables in Table 1, except for corticosteroids, NSAIDs and immunosuppressive agents.  Abbreviation: CI, confidence interval | | | | |
