# Supplementary material for: Arterial cardiovascular outcomes and venous thromboembolism in patients with primary Sjögren’s syndrome: a Danish cohort study
Source: Rheumatology (Oxford). 2025 Apr 23;64(8):4678–86. doi: 10.1093/rheumatology/keaf210 (PMC12316372; doi:10.1093/rheumatology/keaf210)
Supplement: keaf210_Supplementary_Data [file keaf210_supplementary_data.zip › rhe-24-3025-File015.docx]

| **Supplementary Table S9.** Hazard ratios of cardiovascular events in pSS patients compared with the general population cohort. Analyses adjusted for use of drugs used in pSS (corticosteroids, NSAIDs and immunosuppressive agents) within 180 days before the index date. | | | |
| --- | --- | --- | --- |
| **Cardiovascular event** | **Unadjusted hazard ratio (95% CI)*** | **Adjusted hazard ratio (95% CI)**** | **Fully adjusted hazard ratio (95% CI)***** |
| **Myocardial infarction** | 1.32 (1.09 to 1.60) | 1.23 (1.01 to 1.50) | 1.14 (0.94 to 1.40) |
| **Ischaemic stroke** | 1.39 (1.20 to 1.59) | 1.31 (1.14 to 1.52) | 1.29 (1.12 to 1.49) |
| **Haemorrhagic stroke** | 1.56 (1.17 to 2.08) | 1.51 (1.13 to 2.03) | 1.49 (1.10 to 2.01) |
| **Peripheral arterial disease** | 1.50 (1.18 to 1.89) | 1.44 (1.13 to 1.83) | 1.37 (1.07 to 1.74) |
| **Venous thromboembolism** | 1.70 (1.45 to 2.00) | 1.57 (1.33 to 1.85) | 1.47 (1.24 to 1.74) |
| **Heart failure** | 1.34 (1.14 to 1.57) | 1.17 (0.99 to 1.39) | 1.12 (0.94 to 1.33) |
| *Controlled for the matching factors (age, sex, calendar year).  **Controlled for the matching factors by study design and adjusted for the covariables in Table 1, except for corticosteroids, NSAIDs, and immunosuppressive agents.  ***Controlled for the matching factors by study design and adjusted for the covariables in Table 1, as well for drugs used to treat pSS (corticosteroids, NSAIDs, and immunosuppressive agents) as potential mediators or effect modifiers.  Abbreviation: CI, confidence interval | | | |
