# Supplementary material for: Arterial cardiovascular outcomes and venous thromboembolism in patients with primary Sjögren’s syndrome: a Danish cohort study
Source: Rheumatology (Oxford). 2025 Apr 23;64(8):4678–86. doi: 10.1093/rheumatology/keaf210 (PMC12316372; doi:10.1093/rheumatology/keaf210)
Supplement: keaf210_Supplementary_Data [file keaf210_supplementary_data.zip › rhe-24-3025-File016.docx]

| **Supplementary Table S10.** Hazard ratios of cardiovascular events in pSS patients compared with the general population cohort, with the exclusion of covariates, including treatment, that were recorded only within 6 months before the index date. | | | |
| --- | --- | --- | --- |
| **Cardiovascular event** | **Unadjusted hazard ratio (95% CI)*** | **adjusted hazard ratio (95% CI)**** | **Fully adjusted hazard ratio (95% CI)***** |
| **Myocardial infarction** | 1.32 (1.09 to 1.60) | 1.26 (1.03 to 1.53) | 1.20 (0.98 to 1.46) |
| **Ischaemic stroke** | 1.39 (1.20 to 1.59) | 1.34 (1.16 to 1.54) | 1.33 (1.15 to 1.54) |
| **Haemorrhagic stroke** | 1.56 (1.17 to 2.08) | 1.55 (1.16 to 2.08) | 1.49 (1.11 to 2.01) |
| **Peripheral arterial disease** | 1.50 (1.18 to 1.89) | 1.45 (1.14 to 1.84) | 1.37 (1.07 to 1.75) |
| **Venous thromboembolism** | 1.70 (1.45 to 2.00) | 1.60 (1.36 to 1.89) | 1.54 (1.30 to 1.81) |
| **Heart failure** | 1.34 (1.14 to 1.57) | 1.22 (1.04 to 1.44) | 1.19 (1.01 to 1.41) |
| *Controlled for the matching factors (age, sex, calendar year).  **Controlled for the matching factors by study design and adjusted for the covariables in Table 1, except for corticosteroids, NSAIDs, and immunosuppressive agents.  ***Controlled for the matching factors by study design and adjusted for the covariables in Table 1, including corticosteroids, NSAIDs, and immunosuppressive agents.  Abbreviation: CI, confidence interval | | | |
