# Supplementary material for: Arterial cardiovascular outcomes and venous thromboembolism in patients with primary Sjögren’s syndrome: a Danish cohort study
Source: Rheumatology (Oxford). 2025 Apr 23;64(8):4678–86. doi: 10.1093/rheumatology/keaf210 (PMC12316372; doi:10.1093/rheumatology/keaf210)
Supplement: keaf210_Supplementary_Data [file keaf210_supplementary_data.zip › rhe-24-3025-File017.docx]

| **Supplementary Table S11.** Cumulative incidence of cardiovascular events in pSS patients, and hazard ratios compared with the general population cohort, by time period. | | | | |
| --- | --- | --- | --- | --- |
|  | **Cumulative Incidence per 1000 in pSS cohort (95% CI)** | | **Adjusted hazard ratio (95% CI)*** | |
| **Cardiovascular event** | **1996-2002** | **>2002-2016** | **1996-2002** | **>2002-2016** |
| **Myocardial infarction** | 66.19 (51.99 to 82.61) | 21.23 (15.76 to 27.99) | 1.32 (1.02 to 1.71) | 1.10 (0.81 to 1.49) |
| **Ischaemic stroke** | 126.67 (102.24 to 153.82) | 72.25 (56.61 to 90.33) | 1.28 (1.04 to 1.56) | 1.34 (1.09 to 1.64) |
| **Haemorrhagic stroke** | 29.57 (18.91 to 43.96) | 51.23 (20.79 to 102.34) | 1.23 (0.79 to 1.91) | 1.82 (1.21 to 2.72) |
| **Peripheral arterial disease** | 44.46 (31.67 to 60.38) | 19.50 (13.58 to 27.15) | 1.68 (1.20 to 2.35) | 1.28 (0.91 to 1.81) |
| **Venous thromboembolism** | 71.58 (56.38 to 89.10) | 60.91 (46.74 to 77.58) | 1.57 (1.22 to 2.03) | 1.55 (1.24 to 1.93) |
| **Heart failure** | 102.34 (80.59 to 127.04) | 55.53 (37.93 to 77.77) | 1.19 (0.95 to 1.48) | 1.16 (0.91 to 1.50) |
| *Controlled for the matching factors (age, sex, calendar year) by study design and adjusted for the covariables in Table 1 except for corticosteroids, NSAIDs and immunosuppressive agents.  Abbreviation: CI, confidence interval | | | | |
