# Supplementary material for: Arterial cardiovascular outcomes and venous thromboembolism in patients with primary Sjögren’s syndrome: a Danish cohort study
Source: Rheumatology (Oxford). 2025 Apr 23;64(8):4678–86. doi: 10.1093/rheumatology/keaf210 (PMC12316372; doi:10.1093/rheumatology/keaf210)

Supplementary Figure S1. Cardiovascular disease (CVD) occurrence before diagnosis of primary Sjögren’s syndrome (pSS) among the 954 patients presented a CVD before the index date (1-Kaplan Meier plot).


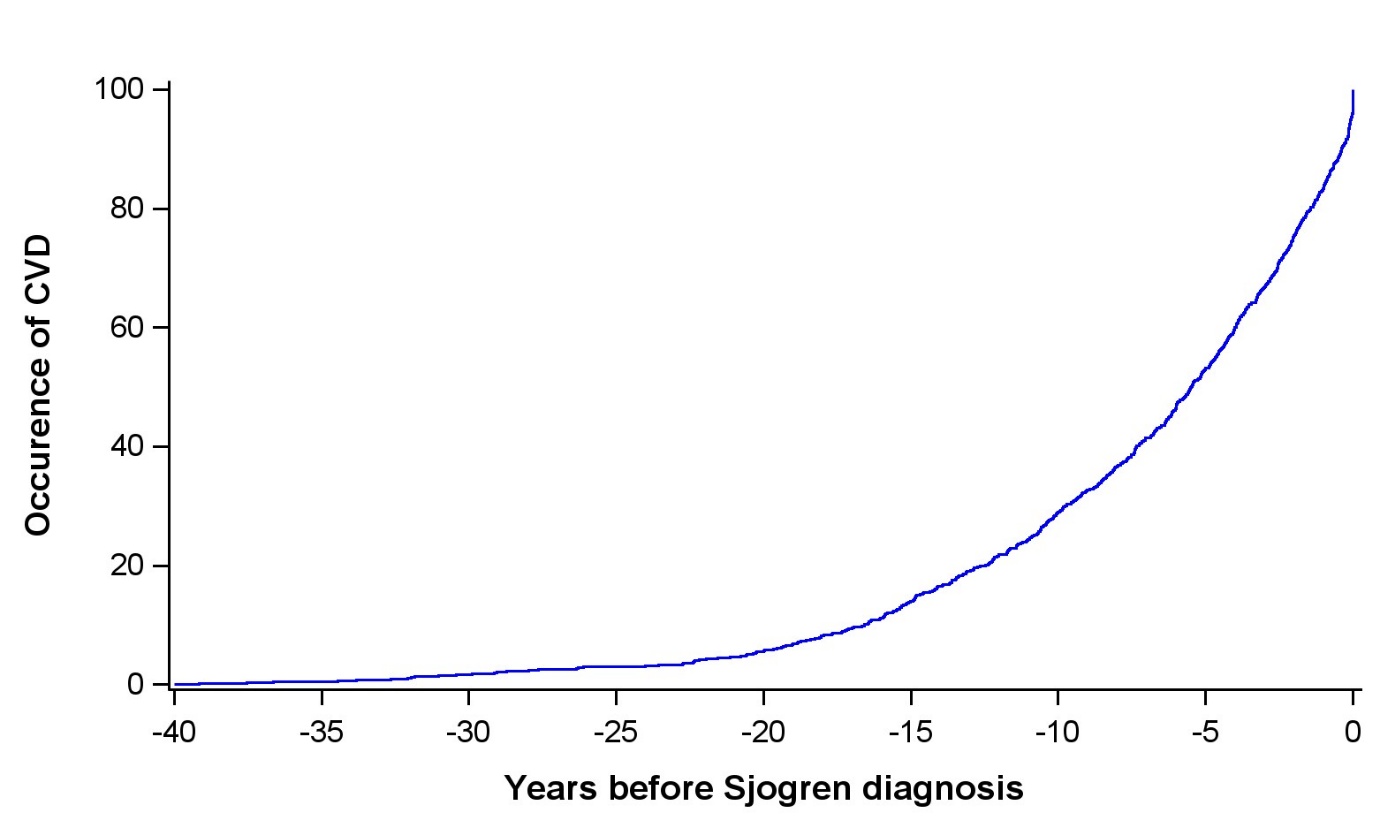

Supplement: keaf210_Supplementary_Data [file keaf210_supplementary_data.zip › rhe-24-3025-File018.docx]
